# Alterations in Mitochondrial Oxidative Stress and Mitophagy in Subjects with Prediabetes and Type 2 Diabetes Mellitus

**DOI:** 10.3389/fendo.2017.00347

**Published:** 2017-12-15

**Authors:** Shipra Bhansali, Anil Bhansali, Rama Walia, Uma Nahar Saikia, Veena Dhawan

**Affiliations:** ^1^Department of Experimental Medicine and Biotechnology, Postgraduate Institute of Medical Education and Research (PGIMER), Chandigarh, India; ^2^Department of Endocrinology, Postgraduate Institute of Medical Education and Research (PGIMER), Chandigarh, India; ^3^Department of Histopathology, Postgraduate Institute of Medical Education and Research (PGIMER), Chandigarh, India

**Keywords:** reactive oxygen species, mitochondrial oxidative stress, mitophagy, prediabetes, type 2 diabetes mellitus

## Abstract

**Background and aim:**

Hyperglycemia-mediated oxidative stress impedes cell-reparative process like autophagy, which has been implicated in impairment of β-cell function in type 2 diabetes mellitus (T2DM). However, the role of mitophagy (selective mitochondrial autophagy) in progression of hyperglycemia remains elusive. This study aimed to assess the impact of increasing severity of hyperglycemia on mitochondrial stress and mitophagy.

**Design and methods:**

A case–control study included healthy controls, subjects with prediabetes, newly diagnosed T2DM (NDT2DM) and advanced duration of T2DM (ADT2DM) (*n* = 20 each). Mitochondrial stress indices, transcriptional and translational expression of mitophagy markers (*PINK1, PARKIN, MFN2, NIX, LC3-II*, and *LAMP-2*) and transmission electron microscopic (TEM) studies were performed in peripheral blood mononuclear cells.

**Results:**

With mild hyperglycemia in subjects with prediabetes, to moderate to severe hyperglycemia in NDT2DM and ADT2DM, a progressive rise in mitochondrial oxidative stress was observed. Prediabetic subjects exhibited significantly increased expression of mitophagy-related markers and showed a positive association with HOMA-β, whereas, patients with NDT2DM and ADT2DM demonstrated decreased expression, with a greater decline in ADT2DM subjects. TEM studies revealed significantly reduced number of distorted mitochondria in prediabetics, as compared to the T2DM patients. In addition, receiver operating characteristic analysis showed HbA_1C_ > 7% (53 mmol/mol) was associated with attenuated mitophagy.

**Conclusion:**

Increasing hyperglycemia is associated with progressive rise in oxidative stress and altered mitochondrial morphology. Sustenance of mitophagy at HbA_1C_ < 7% (53 mmol/mol) strengthens the rationale of achieving HbA_1C_ below this cutoff for good glycemic control. An “adaptive” increase in mitophagy may delay progression to T2DM by preserving the β-cell function in subjects with prediabetes.

## Introduction

Type 2 diabetes mellitus (T2DM) is a chronic metabolic disorder, characterized by insulin resistance and insulin deficiency. Insulin resistance is often the primary metabolic abnormality, which eventually results in progressive decline in β-cell function and emergence of hyperglycemia (1, 2). However, the occurrence of T2DM is preceded by a prolonged phase of prediabetes, which usually lasts for 7–10 years, thereby, providing an opportunity to intervene for the prevention of T2DM. Glucotoxicity, lipotoxicity, increasing oxidative stress, and impaired cell-reparative process, namely autophagy, have been implicated in progressive and inexorable decline in β-cell function/mass (3).

**Graphical AbStract:**
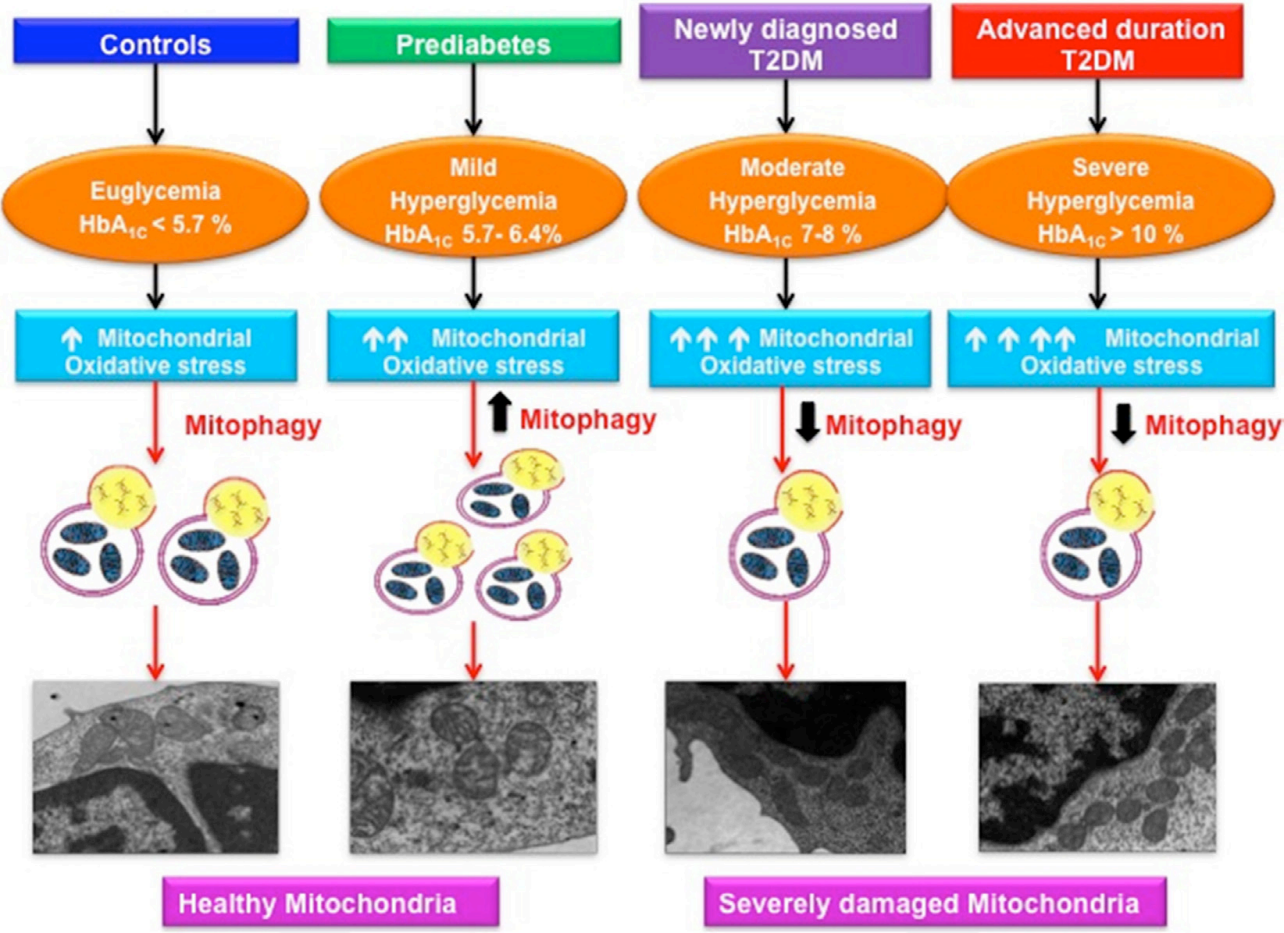
Varying magnitude of mitochondrial oxidative stress and mitophagy in subjects with increasing severity of glycemic burden.

Autophagy is an evolutionary conserved, intracellular catabolic process that involves the sequestration of cellular debris and damaged organelles through the lysosomal machinery. Increasing evidence supports a crucial role of autophagy in the maintenance of β-cell function and survival, as well as insulin sensitivity at target sites including liver, adipocytes, and skeletal muscle (4–6). Consistent with this, β-cell knockdown of Atg7, an autophagic component, resulted in the β-cell loss and accelerated onset of diabetes in rodent models (7, 8). Similarly, a study by Abe et al. (9) reported that insulin-resistant mice exhibited an increased number of autophagosomes in β-cells, indicating autophagic degradation was inhibited in these ob/ob mice. Furthermore, hyperglycemia-induced oxidative stress was shown to be associated with the formation and accumulation of polyubiquitinated protein aggregates in the β-cells of Zucker diabetic fatty rats as a consequence of autophagic dysfunction (10). Regarding studies in patients with T2DM, Abe et al. (9) and Kaniuk et al. (10) reported that autopsy sample of pancreas obtained from these patients showed an increased expression of p62 in the islets, suggesting impaired autophagy.

Recently, mitophagy has emerged as a key facet in maintaining mitochondrial heath and cell survival. Mitophagy, also known as mitochondrial autophagy, involves the selective engulfment of excessive or damaged mitochondria *via* autophagosomes, thereby playing a critical role in regulation of mitochondrial quality and quantity control (11). However, the role of mitophagy in the pathogenesis of T2DM still remains elusive and largely to be explored.

Mitochondria not only play an important role in cellular respiration but are also the primary site for glucose metabolism and endogenous reactive oxygen species (ROS) production. Moreover, mitochondria are also involved in the insulin biosynthesis and secretion as well as in fuel metabolism at insulin target sites. Under physiological conditions, low levels of ROS trigger various cellular-defense mechanisms like upregulation of anti-oxidants and autophagy to protect the cell from oxidative stress-mediated injury. However, at higher levels of ROS, an imbalance in ROS production relative to the cytoprotective action of autophagy results in the accumulation of dysfunctional mitochondria within the cell, thereby, compromising its viability and functionality (12). Furthermore, excessive ROS generation leads to accumulation of mtDNA mutations, which promote the aggregation of “mitophagy-resistant” mitochondria (13). Reports in the literature suggest that defunct mitochondria result in decreased insulin sensitivity and impaired β-cell insulin synthesis and secretion, thus, contributing toward the progression to T2DM (14). Therefore, by understanding the implication of mitophagy in promoting cellular health including β-cell, it may provide a unique insight in identifying the novel therapeutic targets for the treatment of T2DM.

Mitophagy is a genetically regulated process controlled by various genes including PTEN-induced putative kinase 1 (*PINK1*), *PARKIN*, microtubule-associated protein light chain 3 (*LC3*) and lysosome-associated membrane protein-2 (*LAMP-2*), as well as mitophagic receptors NIP3-like protein X (*NIX*) and mitofusin2 (*MFN2*) (15). The sequence of events involved in mitophagy includes flagging of the dysfunctional/damaged mitochondria, *de novo* synthesis of pre-autophagosomal complex around dysfunctional mitochondria, and subsequently sequestration by autophagic machinery. Recognition of damaged mitochondria is facilitated by *NIX* and *PINK1*, leading to recruitment and activation of another molecule *PARKIN*, that catalyzes the ubiquitination of the *MFN2*. These polyubiquitinated proteins serve as a signal for the recruitment of autophagic machinery that involves the autophagosome formation induced by *LC3-II* and its subsequent fusion with lysosome, mediated by another protein *LAMP-2* (16). Thereafter, the engulfed mitochondrial cargo is degraded by lysosomal hydrolases, hence maintaining the mitochondrial health.

We hypothesized that aggravated oxidative stress might be associated with deregulated mitophagy, which in turn may result in worsening of hyperglycemia. Hence, the present study aimed to unravel the magnitude of mitochondrial oxidative stress, mitophagy and the ultra-structural alterations in mitochondrial morphology in individuals with varying severity of hyperglycemia. Peripheral blood mononuclear cells (PBMCs) were opted as a cellular model for assessing the mitochondrial dysfunction in our study as they are the surrogate markers of mitochondrial dysfunction, express insulin receptors and readily respond to circulating glucose and insulin concentration (17). Thus, these cells closely reflect the prevailing physiological milieu at the insulin target sites and β-cells. Furthermore, PBMCs are easily accessible and have been used previously in a couple of studies to demonstrate the alterations in mitochondrial stress indices as well as autophagy (18–20).

## Materials and Methods

### Study Population

Eighty, age, and gender-matched subjects visiting the Endocrinology Clinic of PGIMER, Chandigarh were enrolled. The study was a case–control design and included healthy controls (GP-I), subjects with prediabetes (GP-II), newly diagnosed T2DM (NDT2DM, duration of diabetes < 6 months and drug-naive, GP-III) and patients with advanced duration of T2DM (ADT2DM, duration of diabetes 5–10 years, GP-IV) (*n* = 20 each) with age range 30–55 years and body mass index (BMI) of 25–35 Kg/m^2^. The HbA_1C_ cutoff criteria for healthy controls and subjects with prediabetes was <5.7 and 5.7–6.4%, respectively, whereas, patients of NDT2DM and ADT2DM with HbA_1C_ levels 6.5–8 and >10%, respectively, were recruited for the study. Subjects with abnormal liver and renal function tests and those with a history of coronary artery disease and stroke in the previous 6 months were excluded from the study. All subjects with prediabetes and NDT2DM were drug-naïve, whereas, patients with ADT2DM were on oral hypoglycemic agents including sulfonylureas and metformin, and one-third of the patients were receiving insulin. The study was approved by the Institutional Ethics Committee (IEC) and was performed in accordance with Helsinki Declaration. A written informed consent was obtained from the participants prior to their inclusion in the study.

Detailed anthropometry including height, weight, BMI, waist circumference, and body fat were recorded using standard methods. Study subjects (GP-I–III) were screened for glucose intolerance by standard oral glucose tolerance test using 75 g of anhydrous glucose according to American Diabetes Association (ADA) criteria (21). However, patients in GP-IV, who had an advanced duration of diabetes, and were receiving anti-diabetic medications, underwent fasting and 2-h post-prandial glucose profile as they were receiving anti-diabetic medications. Plasma glucose levels were estimated by glucose oxidase method (Roche autoanalyzer, Germany).

All the experimental procedures were conducted in the fasting state between 0700 and 0800 hours. Subjects with prediabetes and NDT2DM were drug-naïve, while patients with ADT2DM were advised to omit their morning oral anti-diabetic medications 24 h prior to their sampling to abrogate the effect of medications on study parameters. After the completion of baseline workup, all the study subjects were recommended to follow lifestyle modifications including dietary restrictions and physical activity.

### Biochemical Investigations

Biochemical investigations including fasting plasma insulin, C-peptide (Electrochemiluminiscence immunoassay), lipid profile (Autoanalyzer, Roche, Mannheim, Germany) and HbA_1C_ by high-performance liquid chromatography (Bio-Rad D10 system, Hercules, CA, USA), were measured. The insulin resistance and β-cell function were calculated using the homeostasis model assessment HOMA2-IR and HOMA-β, respectively, by the standard formula (22). HOMA2-IR represents insulin sensitivity, whereas HOMA-β is an indicator of β-cell reserve.

### PBMCs Isolation

Approximately 10 ml of blood was collected from the study subjects and PBMCs were isolated using Ficoll–Hypaque (Sigma-Aldrich, St Louis, MO, USA) density gradient centrifugation method (23). The cell pellet was resuspended in 1× phosphate buffer saline (PBS). PBMCs obtained were used for determining the mitochondrial reactive oxygen species (mtROS) production, mitochondrial membrane potential (MMP) and succinate dehydrogenase (SDH) activity. The total cellular RNA, protein extraction, and transmission electron microscopic (TEM) studies were also carried out.

### Assessment of mtROS Content

Peripheral blood mononuclear cells were incubated with 5 µM MitoSOX™ Red reagent (Molecular Probes, Invitrogen CA, USA) (Ex/Em wavelengths: 510/572 nm) for 10 min at 37°C in a humidified 5% CO_2_ incubator and were washed twice with PBS. Autofluorescence of the unstained cells was employed as a negative control for each sample. All the analyses were performed on PBMCs, which were gated based on their morphological characteristics (forward vs. side scatter). Furthermore, mtROS levels were determined using Becton Dickinson FACS Aria II flow cytometer by DIVA software (Becton Dickinson, Franklin Lakes, NJ, USA). The mean fluorescence intensity of the Mitosox-positive cells was calculated by substracting the autofluorescent-positive cells.

### Confocal Analysis

Mitochondrial localization of MitoSox was assessed by confocal microscopy. Briefly, isolated PBMCs were seeded in a 12-well culture plate on coverslips for 24 h at 37°C in a 5% CO_2_ incubator. The cells were washed twice with sterile PBS and were incubated with 5 µM MitoSOX™ Red and Hoechst 33342 (nuclear stain), respectively, for 10 min under similar culture conditions. Furthermore, cells were washed twice in PBS and fixed with 4% paraformaldehydes for 10 min at room temperature. Thereafter, the coverslips were washed with PBS and mounted onto slides with antifade mounting media. The stained cells were observed under the Nikon A1R confocal microscope at 60× magnification.

### Detection of MMP

In the present study, the MMP (ΔΨm) was assessed with 5,5′,6,6′-tetrachloro-1,1′,3,3′-tetraethylbenzimidazolcarbocyanine iodide (JC-1) cationic dye using flow cytometric analysis. JC-1 exhibits potential-driven accumulation in mitochondria, resulting in a fluorescence emission spectral shift from 527 nm (green) to 590 nm (red) (Ex/Em_(green)_/Em_(red)_: 485/527/590 nm). Decreased red fluorescence and corresponding increased green fluorescence suggest depolarized mitochondria.

Peripheral blood mononuclear cells obtained from the study subjects were incubated with 1× JC-1 working solution (1:100 dilution in 1× JC-1 assay buffer) (BD Biosciences, USA) for 10 min suspended in 1× JC-1 assay buffer at 37°C in a humidified 5% CO_2_ incubator under dark conditions. The cells were then washed twice, and the pellet was resuspended in 1× assay buffer for acquisition by FACS. The data were analyzed using the FacsDIVA software (Becton-Dickinson Franklin Lakes, NJ, USA).

### Determination of SDH Activity

Succinate dehydrogenase or Complex-II is an inner mitochondrial membrane-bound enzyme linked to electron transport in the respiratory chain and catalyzes the oxidation of succinate to fumarate in the tricarboxylic acid cycle. SDH is a useful indicator of mitochondrial function as it is a stable enzyme and found at high concentration only in the mitochondria of eukaryotes. Briefly, PBMCs were lysed in hypotonic potassium phosphate buffer (pH 7.5), supplemented with protease inhibitors. Cell lysates were then subjected to sonication and centrifuged at 10,000 rpm for 20 min. The supernatant was collected and protein concentration was estimated by Qubit protein assay kit (Molecular Probes, Invitrogen, CA, USA). Furthermore, the protein samples were pre-incubated with the 1× incubation solution for 2 h at room temperature. Thereafter, the activity buffer containing 2,6-dichloroindophenolate (DCPIP), succinate, ubiquinone, and 40 mM sodium azide (inhibitor of electron transport chain) was added just prior taking the measurements. SDH activity was assessed by monitoring the reduction of 2,6-dichloroindophenolate at 600 nm for 60 min at an interval of 60 s as per manufacturer’s protocol (Abcam, Cambridge, MA, USA). The rate of reaction was calculated in mOD/min.

### RNA Isolation and Real-time Quantitative PCR (RT-PCR)

Total cellular RNA was isolated from the PBMCs by TRIZOL reagent (Life Technologies). The quantity and purity of RNA were determined by measuring the absorbance at 260 nm: 280 nm with a range of 1.8–2 using Nanodrop (Applied Biosystems), and the RNA integrity was confirmed by gel electrophoresis. 2 µg of RNA was reverse transcribed to cDNA template using cDNA synthesis kit (Thermo Fischer scientific, MA, USA) according to the manufacturer’s protocol. All the experiments were carried out in duplicates with two non-template controls as negative controls using human-specific primers on StepOnePlus-real-time PCR system (Applied Biosystems, USA). The sequence of the primers is mentioned in the Table S1 in Supplementary Material. The mRNA levels of *PINK1, PARKIN, NIX, MFN2, LC3-II*, and *LAMP-2* in PBMCs were determined by RT-PCR using SYBR green chemistry (Applied Biosystems). Gene expression was measured as fold change and was evaluated by 2^−ΔCT^ method. The data are represented as relative mRNA expression normalized to human β-actin mRNA expression (24).

### Western Blot Analysis

Cellular protein was extracted using RIPA buffer (1% Triton X-100, 50 mM KCl, 25 mM Hepes, pH 7.8, 10 µg/ml leupeptin, 20 µg/ml aprotinin, 125 µM dithiothreitol, 1 mM Phenylmethanesulfonyl fluoride, and 1 mM sodium orthovanadate), supplemented with protease inhibitors (Cat No. P8340, Sigma-Aldrich, USA) followed by centrifugation at 10,000 rpm for 20 min. The protein concentration in the supernatant samples was determined by Qubit protein assay kit (Molecular Probes, Invitrogen, CA, USA). Cell lysates were resolved on standard SDS-polyacrylamide gel electrophoresis and transferred onto PVDF membranes (Millipore, Bedford, MA, USA) using a semi-dry transfer system (Amersham Biosciences, GE Healthcare, USA). The blots were incubated overnight with primary antibodies against PINK1 (1 µg/ml; Sigma-Aldrich, USA), PARKIN (1 µg/ml), MFN2 (2.51 µg/ml), NIX (0.75 µg/ml), LC3-II (1.5 µg/ml), and LAMP-2 (2 µg/ml), obtained from Abcam (Cambridge, MA, USA), followed by probing with their respective HRP-conjugated secondary antibodies. Blots were scanned using an enhanced chemiluminescence system (Fluorchem M, Protein simple), and the band intensity of target proteins was normalized to β-actin by Image J software (1.47 v).

### Electron Microscopy

Peripheral blood mononuclear cells were fixed with 3% glutaraldehyde in 0.2 M Sorensen’s phosphate buffer (pH 7.4) for 2–4 h at 4°C, post-fixed in 1% OsO_4_ in 0.1 M Millionig’s buffer (pH 7.4) followed by dehydration in 70, 90, 100% alcohol and embedded in epon resin (Agar Scientific, 45359-1EA-F). The blocks were polymerized at 60°C overnight. 50 nm thin sections were stained with aqueous 1% uranyl acetate for 20 min and photographed in a JEM 1400 Plus electron microscope (Jeol Ltd., Tokyo, Japan). Three independent examiners carried out the assessment in 30–35 micrographs from three subjects per group, imaged at 10,000× in a blinded manner and the average number of mitochondria per cell was evaluated. To define the distorted mitochondrial morphology, the following characteristic features such as, individual mitochondrial area, total mitochondrial area per cell (mitochondrial mass), aspect ratio (length of the major axis divided by the length of minor axis of the ellipse equivalent to the mitochondrion), and form factor (perimeter^2^/4π* Area) were measured using Image J software. Aspect ratio represents the mitochondrial length, while form factor indicates the mitochondrial branching. In addition, degeneration of cristae and matrix were also taken into consideration for calculating the percentage of distorted mitochondria.

### Statistical Analysis

All data are expressed as mean ± SD and median and interquartile range. For normally distributed data, means of four groups were compared using one-way ANOVA followed by *post hoc* Bonferroni test. For skewed continuous data, Kruskal–Wallis test was used. Receiver operating characteristic (ROC) curves were plotted using sensitivity vs. 1-specificity to obtain the maximal cutoff values of HbA_1C_ for expression of mitophagy-related markers by SVM method. Spearman and Pearson correlation coefficient was calculated to see relationship between different variables. Values with *p* < 0.05 were considered statistically significant. Regression analysis was carried out to find independent predictors of HOMA-β. The statistical analysis was performed using the SPSS version 22 software for window (SPSS Inc., Chicago, IL, USA).

## Results

### Clinical and Biochemical Characteristics of the Study Subjects

The glycemic parameters were consistent with the ADA criteria (21). HOMA2-IR, a marker of insulin resistance, was significantly higher in patients with NDT2DM and ADT2DM as compared to the controls (*p* < 0.05), whereas HOMA-β indices, marker of β-cell function, were significantly reduced in subjects with NDT2DM and ADT2DM vs. controls and prediabetes (*p* < 0.05). ADT2DM subjects had lower LDL-C levels as compared to the subjects with prediabetes and NDT2DM (*p* < 0.05), as they were on statins (Table 1).

**Table 1:** Clinical and biochemical characteristics of the study subjects.

Parameters	Controls (Gp I) (*n* = 20)	Subjects with prediabetes (Gp II) (*n* = 20)	Subjects with NDT2DM (Gp III) (*n* = 20)	Subjects with ADT2DM (5–10 years) (Gp IV) (*n* = 20)
Age (years)	42.9 ± 7.7	43.0 ± 7.9	45.7 ± 9.7	44.8 ± 7.05
Gender (M:F)	10:10	7:13	11:9	10:10
Height (cm)	161.4 ± 10.4	158.9 ± 6.8	158.3 ± 8.9	159.9 ± 10.3
Weight (kg)	73.2 ± 9.3	77.1 ± 9.9	73.1 ± 8.9	75.5 ± 11.0
Body mass index (kg/m^2^)	28.1 ± 2.3	30.4 ± 3.0	29.4 ± 3.3	29.4 ± 2.5
WC (cm)	95.7 ± 8.7	98.0 ± 8.7	98.6 ± 8.0	98.1 ± 8.6
Body fat (%)	30.2 ± 7.3	34.9 ± 6.5	34.2 ± 10.4	33.6 ± 6.7
Systolic BP (mm/Hg)	119.7 ± 12.1	122.2 ± 11.8	126.2 ± 15.2	129.8 ± 16.6
Diastolic BP (mm/Hg)	80.7 ± 7.9	85.1 ± 8.9	81.8 ± 9.6	87.1 ± 8.4
FPG (mmol/l)	5.0 ± 0.6	6.1 ± 0.6	8.6 ± 1.6^***#^	11.4 ± 3.6^***###^
2hrPG (mmol/l)	6.6 ± 1.0	9.1 ± 1.8	13.2 ± 3.0^***#^	15.8 ± 3.6^***###^
HbA_1c_ (%)	5.3 ± 0.2	6.1 ± 0.2*	7.7 ± 0.5^***#^	11.6 ± 1.3^***###$^
HbA_1c_ (mmol/mol)	34.4 ± 0.2	43.2 ± 0.2*	60.7 ± 0.5^***#^	103.3 ± 1.3^***###$^
FCP (nmol/l)	0.8 ± 0.4	1.0 ± 0.7	1.1 ± 0.3*	0.9 ± 0.4
HOMA2-IR	1.8 ± 0.8	2.4 ± 0.6	2.8 ± 1.0**	2.8 ± 1.3**
HOMA-β	147.3 ± 50.5	120.3 ± 35.0	68.3 ± 18.6^***##^	40.8 ± 24.4^***###^
S. CHOL (mg/dl)	181.8 ± 40.1	207.2 ± 31.7	203.1 ± 41.9	146.2 ± 41.7^##$$^
S. LDL-C (mg/dl)	107.1 ± 33.2	139.7 ± 31.9	136.9 ± 41.9	86.2 ± 43.1^##$$^
S. TG (mg/dl)	139.4 ± 79.4	174.2 ± 54.6	152.4 ± 57.5	147.2 ± 86.9
S. HDL-C (mg/dl)	53.1 ± 17.7	44.1 ± 10.7	44.5 ± 6.7	46.7 ± 13.8

*Data are expressed as mean ± SD*.

*p < 0.05, **p < 0.01, ***p < 0.001 vs. controls.

*^#^p < 0.05, ^##^p < 0.01, ^###^p < 0.001 vs. prediabetic subjects*.

*^$^p < 0.05, ^$$^p < 0.01 vs. NDT2DM patients*.

### mtROS Content

**Figure 1:**
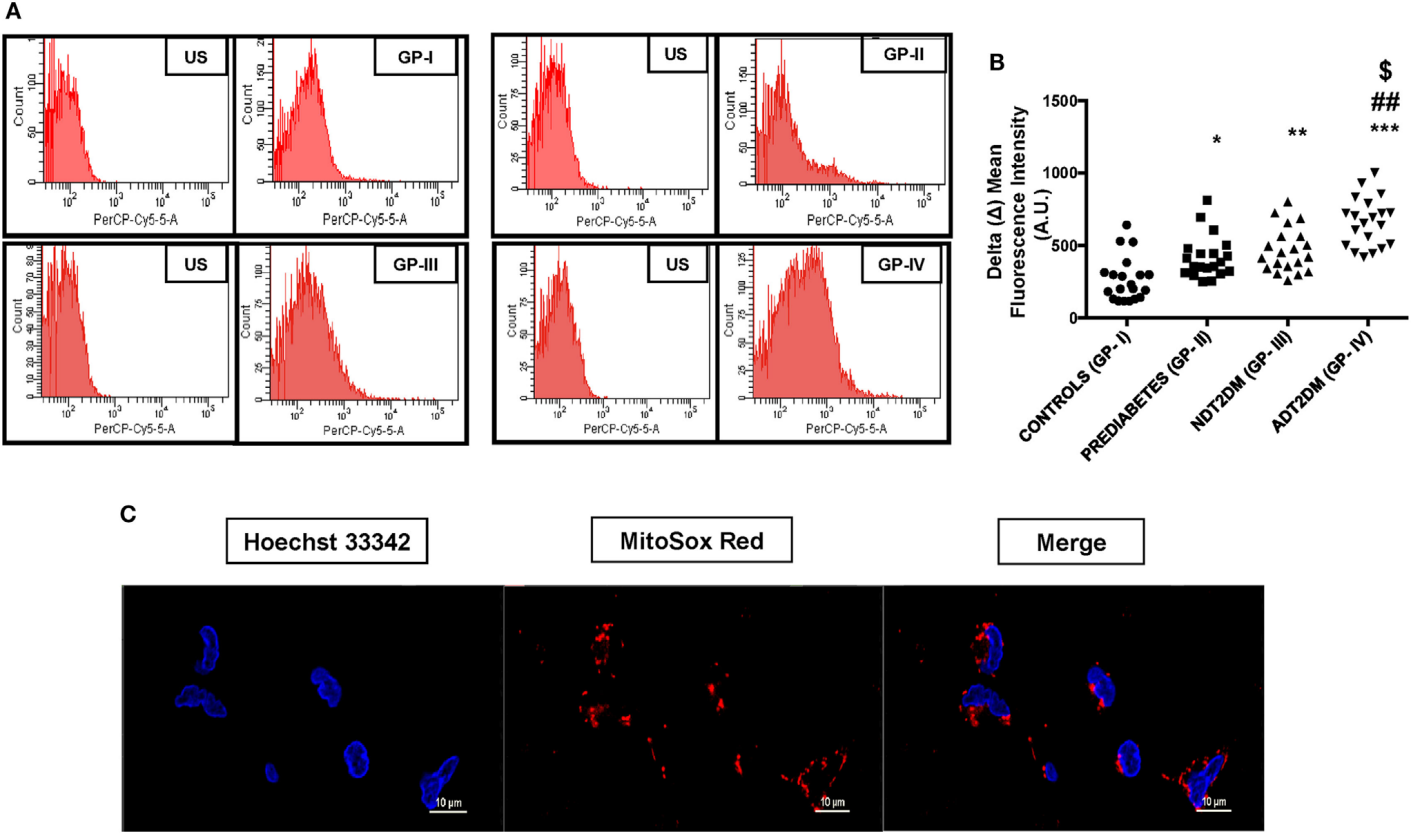
Depicts mitochondrial reactive oxygen species (mtROS) content in study subjects. US-unstained (autofluorescence). **(A)** Representative FACS image of mitosox fluorescence intensity **(B)** bar graph represents delta mean fluorescence in controls (GP-I); prediabetic subjects (GP-II); NDT2DM (GP-III); and ADT2DM patients (GP-IV). Values are expressed in median and interquartile range (*n* = 20). (* = vs. GP-I), (^#^ = vs. GP-II), (^$^ = vs. GP-III), *p < 0.05, **p < 0.01; ***p < 0.001; ^##^*p* < 0.01; ^$^*p* < 0.05. **(C)** Confocal microscopy in human peripheral blood mononuclear cells shows MitoSOX (red fluorescence) localization in the mitochondria, but not in the nucleus. Hoechst 33342 (blue fluorescence) was used for nuclear staining (magnification: 60×).

Mitochondrial reactive oxygen species levels were significantly higher in subjects with prediabetes, NDT2DM and ADT2DM as compared to the controls (*p* < 0.05). Moreover, mtROS content was maximally increased in ADT2DM subjects as compared to prediabetes and NDT2DM patients (*p* < 0.05) (Figures 1A–C).

### Assessment of MMP

**Figure 2:**
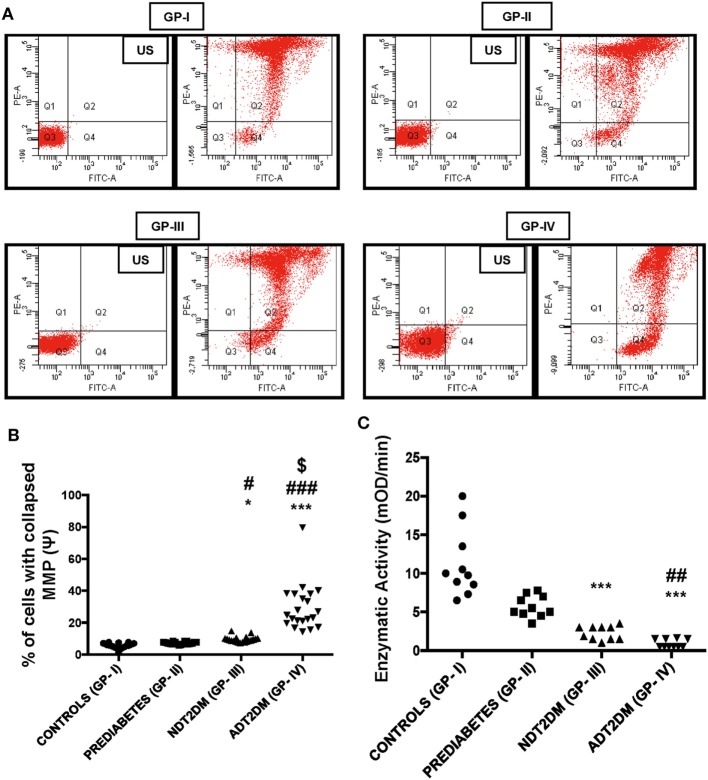
Mitochondrial membrane potential (MMP) and succinate dehydrogenase (SDH) activity in the study subjects. US-unstained (autofluoroscence) **(A)** representative dot-plot of MMP **(B)** bar diagram displaying percentage of cells with collapsed MMP (*n* = 20 each in all the four groups). The percentage of cells with collapsed MMP in type 2 diabetes mellitus (T2DM) patients is higher relative to controls as measured by the green fluorescence intensity (Q4). Increased JC-1 green fluorescence is indicative of mitochondrial membrane depolarization. **(C)** Bar diagram showing SDH enzymatic activity in control (GP-I); prediabetic subjects (GP-II); NDT2DM (GP-III); and ADT2DM patients (GP-IV). Values are expressed in median and interquartile range (*n* = 10). (* = vs. GP-I), (^#^ = vs. GP-II), (^$^ = vs. GP-III), *p < 0.05, ***p < 0.001; ^#^*p* < 0.05; ^##^*p* < 0.01; ^###^*p* < 0.001; ^$^*p* < 0.01.

The percentage of cells with collapsed MMP in PBMCs was found to be significantly higher in patients with NDT2DM vs. controls and prediabetic group (*p* < 0.05). Moreover, the MMP was maximally collapsed in ADT2DM subjects as compared to the other study groups (*p* < 0.05) (Figures 2A,B).

### SDH Activity

Succinate dehydrogenase activity was remarkably reduced in NDT2DM and ADT2DM subjects vs. controls (*p* < 0.05). Similarly, it was also significantly decreased in ADT2DM subjects as compared to the subjects with prediabetes (*p* < 0.05), whereas no significant difference was observed in prediabetic subjects as compared to the healthy controls (Figure 2C).

### Correlation between Mitochondrial Stress Indices and HbA_1C_ Levels

In prediabetic subjects, a positive and significant correlation was observed between HbA_1C_ and mtROS levels (*r* = 0.805; *p* < 0.01). In patients with T2DM, the severity of mitochondrial oxidative stress was more with rising HbA_1C_ levels as reflected by MMP (*r* = 0.880; *p* < 0.01), mtROS levels (*r* = 0.916; *p* < 0.01), and SDH activity (*r* = −0.874, *p* < 0.01).

### Transcriptional Profiling of Mitophagy-Related Genes

**Figure 3:**
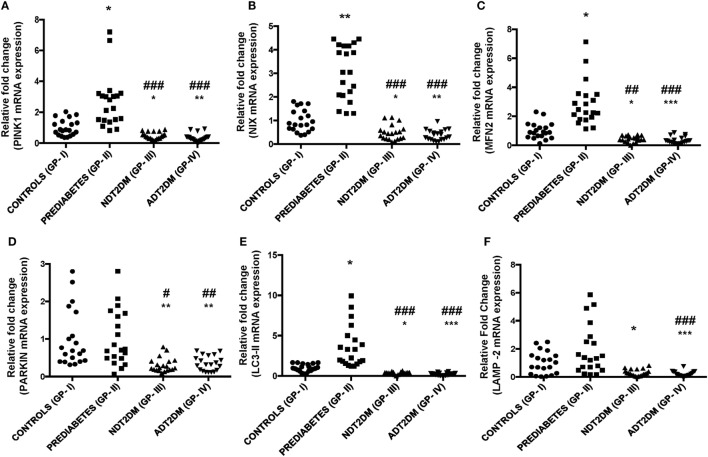
Depicts mRNA expression of mitophagy-related genes in the study subjects with varying degree of glycemic burden. Real-time qPCR analysis of **(A)** *PINK1* **(B)** *NIX* **(C)** mitofusin2 (*MFN2*) **(D)** *PARKIN* **(E)** *LC3-II* **(F)** lysosome-associated membrane protein-2 (*LAMP-2*) in controls (GP-I); prediabetic subjects (GP-II); NDT2DM (GP-III); and ADT2DM patients (GP-IV). Values are expressed in median and interquartile range (*n* = 20). (* = vs. GP-I), (^#^ = vs. GP-II), *p < 0.05; **p < 0.01; ***p < 0.001; ^#^*p* < 0.05; ^##^*p* < 0.01; ^###^*p* < 0.001.

*PINK1* and *NIX* mRNA expression were significantly augmented by ~3- to 4-folds in subjects with prediabetes as compared to the controls (*p* < 0.05). However, *PINK1* and *NIX* gene expression were observed to be significantly attenuated in NDT2DM and ADT2DM subjects vs. controls (*p* < 0.05). A similar trend in the expression of these genes was also observed in NDT2DM and ADT2DM subjects as compared to the prediabetic subjects (*p* < 0.05) (Figures 3A,B).

A significant increase in *MFN2* and *LC3-II* gene expression was observed in subjects with prediabetes vs. controls (*p* < 0.05), while, *PARKIN* showed a non-significant increase in the prediabetic group. However, mRNA expression of *MFN2, PARKIN*, and *LC3-II* were found to be significantly decreased in subjects with NDT2DM and ADT2DM as compared to the controls and subjects with prediabetes (*p* < 0.05) (Figures 3C–E). As far as *LAMP-2* mRNA expression is concerned, it was insignificantly higher in prediabetic group vs. controls. However, a significant reduction in *LAMP-2* mRNA levels was observed in NDT2DM and ADT2DM patients as compared to the healthy controls and prediabetic subjects (*p* < 0.05) (Figure 3F).

### Correlation between mRNA Expression of Mitophagy-Markers and HbA_1C_ Levels

In subjects with prediabetes, *MFN2* (*r* = 0.558; *p* < 0.01), *PINK1* (*r* = 0.534; *p* < 0.01), *NIX* (*r* = 0.637; *p* < 0.01) and *LC3-II* (*r* = 0.667; *p* < 0.01) mRNA expression was significantly and positively correlated with the HbA_1C_ levels. However, in patients with diabetes, a significant and negative correlation was observed between HbA_1C_ levels and mitophagy-related genes including *PINK1* (*r* = −0.563; *p* < 0.01), *MFN2* (*r* = −0.651; *p* < 0.01), *NIX* (*r* = −0.581, *p* < 0.01), *PARKIN* (*r* = −0.518; *p* < 0.01), *LC3-II* (*r* = −0.665; *p* < 0.01), and *LAMP-2* (*r* = −0.744, *p* < 0.01).

### Translational Profiling of Mitophagy-Markers

**Figure 4:**
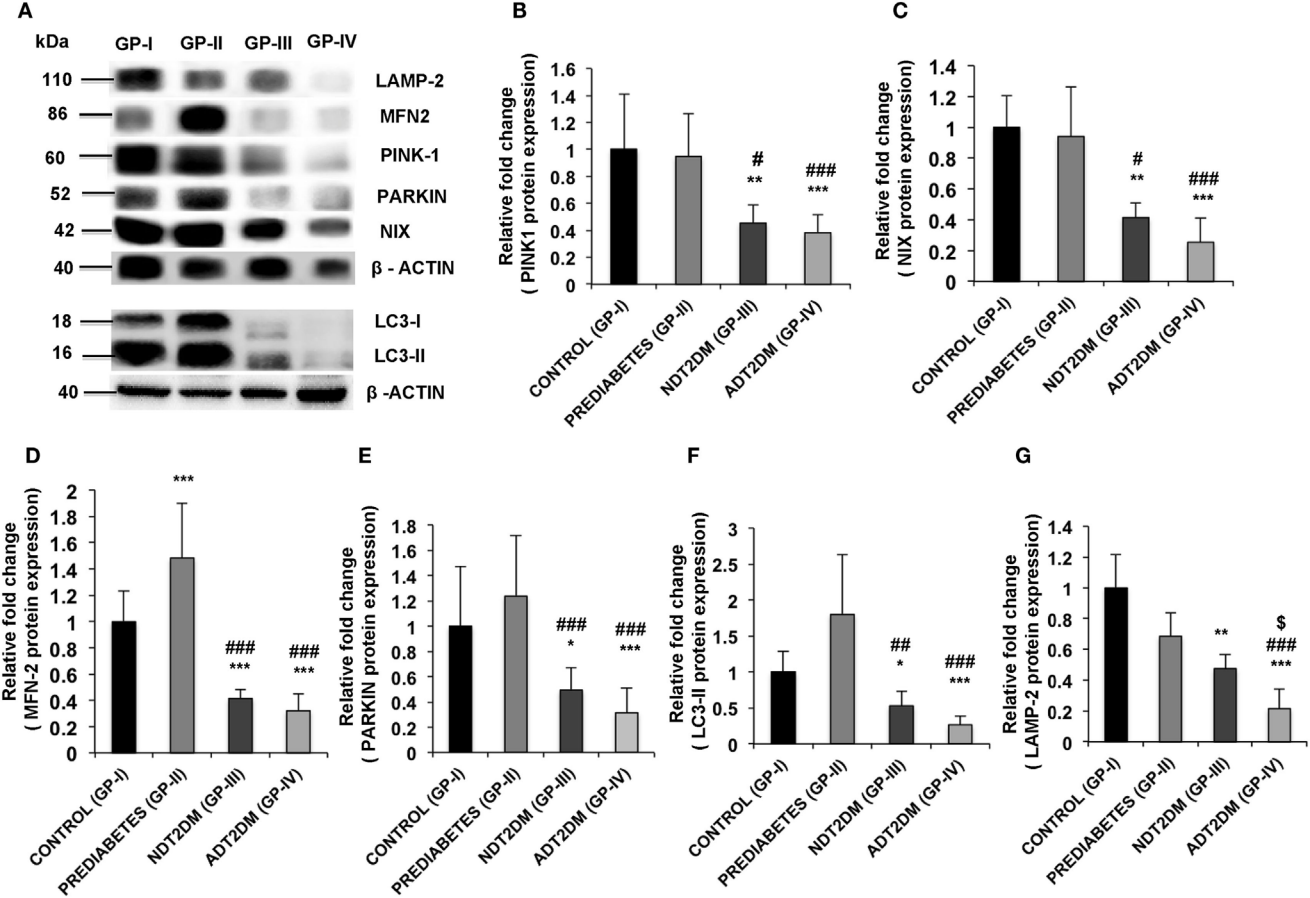
**(A)** Western blot analysis of mitophagy-related markers and quantification of western blots for protein expression of **(B)** PINK1 **(C)** NIX **(D)** mitofusin2 (MFN2) **(E)** PARKIN **(F)** LC3-II **(G)** lysosome-associated membrane protein-2 (LAMP-2) in controls (GP-I); prediabetic subjects (GP-II); NDT2DM (GP-III); and ADT2DM patients (GP-IV). Values are expressed in mean ± SD; (*n* = 10). (* = vs. GP-I), (^#^ = vs. GP-II), (^$^ = vs. GP-III), *p < 0.05; **p < 0.01; ***p < 0.001; ^#^*p* < 0.05; ^##^*p* < 0.01; ^###^*p* < 0.001, ^$^*p* < 0.05.

A significant increase in MFN2 expression was observed in subjects with prediabetes relative to the controls (*p* < 0.05). However, protein expression of PINK1 and NIX was comparable between the two groups. Furthermore, PARKIN and LC3-II expression was insignificantly higher, whereas, LAMP-2 protein levels were insignificantly decreased in prediabetic subjects as compared to the controls. In patients with NDT2DM and ADT2DM, a significant decline in the expression of PINK1, MFN2, NIX, PARKIN, and LC3-II proteins was observed as compared to the controls and prediabetic subjects (*p* < 0.05). Regarding LAMP2 protein expression, it was significantly downregulated in NDT2DM patients vs. controls (*p* < 0.05) and ADT2DM patients vs. all the other study groups (*p* < 0.05) (Figures 4A–G).

### Correlation between Protein Expression of Mitophagy-Markers and HbA_1C_ Levels

In subjects with prediabetes, MFN2 expression (*r* = 0.438; *p* = 0.05) showed a positive correlation with HbA_1C_ levels. However, in patients with diabetes, a significant and negative correlation was observed between rising HbA_1C_ levels and mitophagy markers including PINK1 (*r* = −0.739; *p* < 0.01), MFN2 (*r* = −0.734; *p* < 0.01), NIX (*r* = −0.806, *p* < 0.01), PARKIN (*r* = −0.730; *p* < 0.01), LC3II (*r* = −0.709; *p* < 0.01), and LAMP2 (*r* = −0.896, *p* < 0.01).

### ROC Analysis

**Figure 5:**
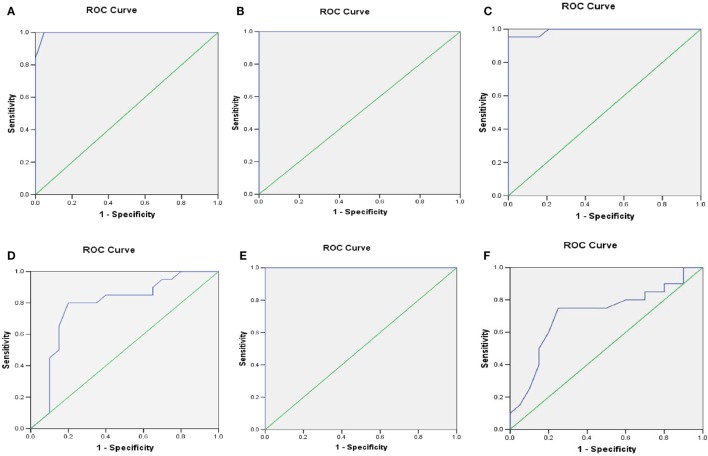
Receiver operating characteristic (ROC) curve was plotted using sensitivity and specificity with area under the curve (AUC) (95% CI) **(A)** *PINK1* **(B)** mitofusin2 (*MFN2*) **(C)** *NIX* **(D)** *PARKIN* **(E)** *LC3-II* **(F)** *LAMP-2* transcriptional levels for predicting the HbA_1C_ cutoff value crucial for the sustenance of mitophagy.

At HbA_1C_ 7%, the area under the curve (AUC) for *MFN2, NIX, PARKIN, PINK1, LC3-II*, and *LAMP-2* was more than 70%. The optimal sensitivity and specificity achieved for *MFN2* at HbA_1C_ 7% were 100 and 90%, *NIX* (95 and 90%), *PARKIN* (80 and 70%), *PINK1* (100 and 86%), respectively. Similarly, for the autophagy markers, *LC3-II*, the sensitivity was 100% and specificity 90%, and for *LAMP-2*, 75 and 65%, respectively (Table 2; Figures 5A–F).

**Table 2:** Sensitivity and specificity of different mitophagy markers at HbA_1C_ cutoff 7% by receiver operating characteristic (ROC) analysis.

Variables	Cutoff value of HbA_1C_ (%)	AUC (95% CI) (*P* value)	Sensitivity (%)	Specificity (%)
Mitofusin2 (MFN2) mRNA levels	7	1.000 (1.000–1.000) (*p* < 0.001)	100	90
NIX mRNA levels	7	0.991 (0.971–1.012) (*p* < 0.0001)	95	90
PARKIN mRNA levels	7	0.781 (0.628–0.934) (*p* < 0.002)	80	70
PINK1 mRNA levels	7	0.996 (0.985–1.000) (*p* < 0.0001)	100	86
LC3-II mRNA levels	7	1.000 (1.000–1.000) (*p* < 0.0001)	100	90
Lysosome-associated membrane protein-2 (LAMP-2) mRNA levels	7	0.714 (0.547–0.880) (*p* < 0.02)	75	65

*AUC, area under curve*.

### Regression Analysis to Determine the Association between HOMA-β and Mitophagy Markers

In the control group, the variation in HOMA-β indices contributed by mitophagy-related genes (*PINK1, MFN2, NIX*, and *LC3-II*) was observed to be only 16.9%, which was statistically non-significant, whereas in subjects with prediabetes, variation in HOMA-β associated with these genes was 60.6%, which was highly significant (*p* = 0.005).

### Quantification of Mitochondrial Morphological Characteristics

**Figure 6:**
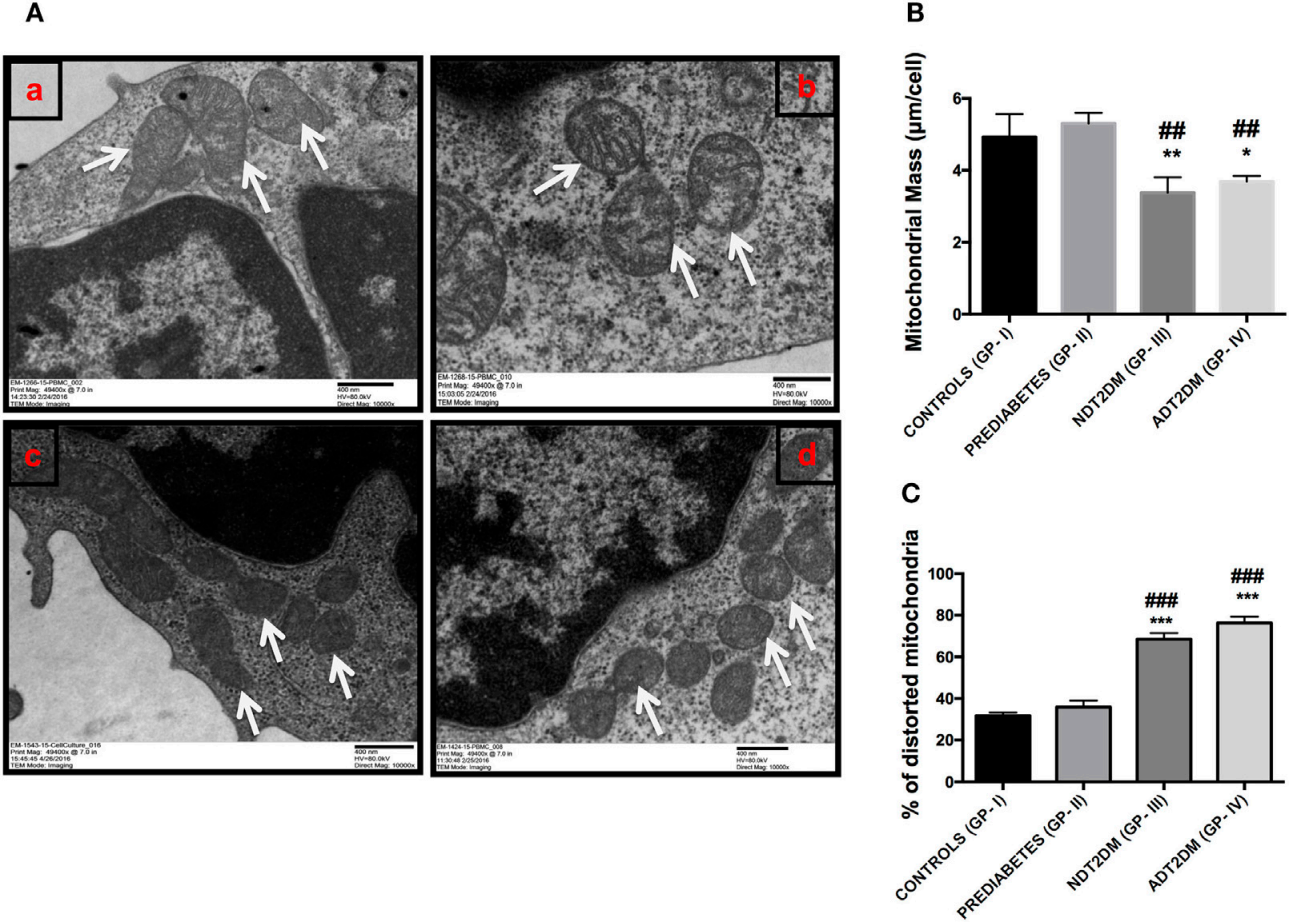
**(A)** Representative electron micrographs of mitochondria in the study subjects (Original magnification 10,000×, print magnification 49,400×@7.in 400 nm scale). White arrows represent mitochondria. The panels indicating (a) Controls (healthy and larger mitochondria) (GP-I); (b) Prediabetic subjects (healthy and larger mitochondria) (GP-II); (c) NDT2DM patients (GP-III), and (d) ADT2DM patients (GP-IV). Healthy mitochondria were characterized by oval or spherical shape, well-defined cristae and dense matrix, while mitochondria in type 2 diabetes mellitus (T2DM) subjects were smaller in size, rounded and with degenerated matrix **(B)** bar diagram displaying the total mitochondrial area per cell in all the study groups **(C)** bar graph represents the percentage of distorted mitochondria in controls (GP-I); prediabetic subjects (GP-II); NDT2DM patients (GP-III); ADT2DM patients, who were randomly selected with similar clinical and biochemical profile in each group *(n* = 3). Values are expressed in mean ± SD; (* = vs. GP-I) (^#^ = vs. GP-II), *p < 0.05; **p < 0.01, ***p < 0.001; ^##^*p* < 0.01; ^###^*p* < 0.001.

The details of mitochondrial morphology are mentioned in Table 3. No significant difference was observed in the mitochondrial number per cell among all the study groups. The average individual mitochondria area (μm) and total mitochondrial area or mass per cell (μm/cell) were comparable between the controls and prediabetic group, whereas, it was significantly reduced in patients with NDT2DM and ADT2DM as compared to the controls and subjects with prediabetes. Furthermore, in subjects with prediabetes, there was a decreasing trend toward the aspect ratio and degree of mitochondrial branching complexity as measured by the form factor, though it did not reach statistical significance. However, the aspect ratio and mitochondrial branching was significantly reduced in patients with NDT2DM and ADT2DM as compared to the controls. Based on these parameters, as well as the inclusion of degenerated matrix and cristae, the percentage of distorted mitochondria in subjects with prediabetes was observed to be comparable with the controls, whereas, it was significantly increased in patients with NDT2DM and ADT2DM (*p* < 0.05) (Table 3; Figures 6A–C).

**Table 3:** Details of mitochondrial morphology.

Morphological characteristics	Controls (GP-I)	Prediabetes (GP-II)	NDT2DM subjects (GP-III)	ADT2DM subjects (GP-IV)
Mitochondria (#/cell)	8.1 ± 1.5	7.1 ± 0.9	7.3 ± 0.6	7.7 ± 1.9
Average individual mitochondrial area (μm)	0.60 ± 0.1	0.74 ± 0.04	0.50 ± 0.01^*#^	0.48 ± 0.03^*#^
Total mitochondrial area/Cell (μm/cell)	4.92 ± 0.9	5.30 ± 0.42	3.68 ± 0.60^**#^	3.68 ± 0.22^**##^
Aspect ratio	1.90 ± 0.9	1.62 ± 0.9	1.29 ± 0.4**	1.29 ± 0.2**
Form factor	1.65 ± 0.7	1.37 ± 0.5	1.17 ± 0.3**	1.17 ± 0.1**
% of distorted mitochondria	31.7 ± 2.2	35.5 ± 4.4	68.4 ± 4.2^***###^	76.6 ± 4.4^***###^

Data is expressed as mean ± SD, (* = vs. controls) (^#^ = vs. prediabetic subjects); *p < 0.05, **p < 0.01,***p < 0.001, ^#^*p* < 0.05, ^##^*p* < 0.01, ^###^*p* < 0.001.

### Correlation between the Percentage of Distorted Mitochondria and Mitophagy-Markers

A significant and negative correlation was observed between the percentage of distorted mitochondria and mitophagy-related genes including *PINK1* (*r* = −0.603; *p* = 0.038), *MFN2* (*r* = −0.598; *p* = 0.040), *NIX* (*r* = −0.596, *p* = 0.041), and *PARKIN* (*r* = −0.601; *p* = 0.039). However, for the classical autophagy markers like *LC3-II* (*r* = −0.529; *p* = 0.077) and *LAMP-2* (*r* = −0.559, *p* = 0.059), an insignificant inverse correlation was observed.

## Discussion

The present study demonstrates that with increasing severity of hyperglycemia, there is a progressive rise in mitochondrial oxidative stress indices, as evidenced by enhanced mtROS content, reduced MMP and SDH activity. Subjects with prediabetes accompanied by mild hyperglycemia, exhibited an adaptive increase in mitophagy-related markers and mitochondrial mass, whereas, patients with T2DM displayed attenuated expression of these markers and reduced mitochondrial mass. Furthermore, augmented mitophagy in prediabetic group was associated with decreased mitochondrial damage as shown by TEM studies, while, T2DM patients who exhibited reduced mitophagy had increased mitochondrial damage. In addition, HbA_1C_ > 7% (53 mmol/mol) was associated with progressive decline in mitophagy irrespective of duration of diabetes, hence, strengthening the rationale of achieving HbA_1C_ below this threshold for good glycemic control at any stage of the disease.

Alterations in mtROS, MMP, and SDH activity are the surrogate markers of mitochondrial dysfunction. To the best of our knowledge, this is the first study demonstrating the impact of varying glycemic burden on the magnitude of mitochondrial stress indices. Subjects with prediabetes, patients with NDT2DM and ADT2DM showed a progressive rise in oxidative stress with increasing HbA_1C_ levels. Though, no significant alterations in MMP and SDH activity were observed in subjects with prediabetes as compared to the controls, indicating that mild hyperglycemia in prediabetic subjects resulted in a modest rise in mitochondrial oxidative stress levels. Furthermore, a positive correlation between mitochondrial oxidative stress indices and increasing HbA_1C_ levels suggests that increasing glycemic burden is accompanied with enhanced mitochondrial dysfunction in patients with T2DM.

It is imperative to note that the magnitude of expression of mitophagy-related genes has not been explored in association with the increasing severity of glycemic burden. Our study is the first report to demonstrate that *MFN2, NIX, PINK1*, and *PARKIN* expression was increased in subjects with prediabetes as compared to the healthy controls. At translational level, though NIX and PINK1 expression was comparable to the controls, MFN2 expression showed a significant increase. Therefore, it is appropriate to state that in prediabetic subjects, mild hyperglycemia-mediated oxidative stress upregulates mitophagy, which is an adaptive response and an important coping mechanism required to maintain cell survival by eliminating the dysfunctional mitochondria, thereby limiting the mitochondrial oxidative stress. Our observations are further supported by another study, which demonstrated that mild oxidative stress triggers mitophagy in a Drp-1 dependent manner to promote cell survival (25). Moreover, “adaptive” increase in mitophagy showed a positive association with HOMA-β indices, suggesting that the upregulated mitophagy in prediabetic subjects may delay the progression to T2DM by preserving the β-cell function.

Furthermore, a significant downregulation in the mRNA and protein expression of MFN2, NIX, PINK1, and PARKIN was observed in NDT2DM and ADT2DM subjects. This suggests that moderate to severe hyperglycemia-mediated oxidative stress attenuates the expression of these genes and consequently impairs mitophagy, resulting in accumulation of dysfunctional mitochondria and increased ROS generation. Our observations are consistent with another study, which demonstrated reduced MFN2 expression in retina of diabetic rats and human donors with duration of diabetes ≥ 10 years (26). Furthermore, Scheele et al. (27) reported a significant downregulation of *PINK1* transcripts in skeletal muscle biopsies obtained from T2DM subjects. Recently, a study showed reduced mRNA expression of *MFN2, PARKIN*, and *PINK1* in patients with diabetic nephropathy, which corroborates with our observations (19).

In addition to the mitophagy markers, we also examined the classical autophagy markers like LC3-II and LAMP-2. Subjects with prediabetes showed an increase in *LC3-II* and *LAMP-2* mRNA expression which is consistent with the observations of Llopis et al. (28) who demonstrated augmented LC3-II protein expression in T2DM patients with HbA_1C_ 6.8% (50.8 mmol/mol) similar to prediabetic subjects in our study with HbA_1C_ 6% (42.1 mmol/mol). This suggests that the ambient glycemic burden is an important denominator to influence the process of mitophagy, rather than the duration of diabetes.

A significant reduction in both LC3-II and LAMP-2 mRNA and protein expression was observed in subjects with NDT2DM and ADT2DM. A recent study by Moller et al. (29) reported that the insulin resistant patients with T2DM displayed reduced LC3-II expression in skeletal muscles as compared to the control subjects. However, Llopis et al. (28) observed augmented LC3-II protein expression in diabetic patients. The discrepancy in these observations may be attributed to variability in severity of hyperglycemia, as the mean HbA_1C_ was 7.7% (60.7 mmol/mol) and 11.6% (103.3 mmol/mol) in NDT2DM and ADT2DM patients, respectively, in our study as compared to 6.8% (50.8 mmol/mol) in the former. Furthermore, Liu et al. (30) demonstrated attenuated *LAMP-2* expression resulted in reduced autophagy in rat islets, leading to β-cell dysfunction, which is similar to our observations. Interestingly, LAMP-2 expression was significantly attenuated in patients with ADT2DM as compared to NDT2DM, suggesting that aggravated oxidative stress may impede the terminal event in mitophagy, thereby, resulting in further deterioration of cellular health.

Reports in the literature suggest that increased oxidative stress induces PINK1 accumulation and subsequent translocation of PARKIN to the damaged mitochondria (31). However, our study demonstrated a decreased expression of PINK1 and PARKIN in T2DM patients, which can be explained by the fact that the whole cellular lysates were assessed rather than the mitochondrial extracts. Nonetheless, our data revealed that the rising oxidative stress in T2DM patients impairs the terminal event of PINK1 and PARKIN-mediated mitophagy, as depicted by the reduced expression of LC3-II and LAMP2 proteins, consequently leading to concomitant accumulation of the superfluous mitochondria.

Despite similar magnitude of mitochondrial oxidative stress amid subjects with prediabetes and NDT2DM patients, there was a marked variability in the expression levels of mitophagy-related genes between the two groups. This indicates that besides ROS-mediated damage, glucotoxicity *per se* may influence the mitophagic process in NDT2DM subjects, as substantiated by negative correlation between HbA_1C_ levels and mitophagy markers in these patients. Our findings are strengthened by an *in vitro* study, which demonstrated that INS-1 cells, when cultured in the presence of low glucose concentration (10 mM), akin to the glucose milieu in prediabetes, activate AMPK, which in turn induces autophagy (32). However, at a higher concentration of glucose (30 mM), mimicking the uncontrolled hyperglycemic conditions in T2DM subjects, autophagy was inhibited via mTOR activation as shown in cardiac myocytes (33).

Transmission electron microscopic analysis revealed non-significant alterations in the percentage of distorted mitochondria as indicated by total mitochondrial area, aspect ratio and form factor, in subjects with prediabetes relative to controls. These observations provide compelling evidence that increased mitophagy in subjects with prediabetes may result in elimination of dysfunctional mitochondria, thereby, preventing their accumulation and further aggravation of mitochondrial oxidative stress. On the contrary, patients with T2DM had markedly increased ROS levels, which not only induced an increase in mitochondrial damage but also suppressed mitophagy, thereby, resulting in enhanced accumulation of damaged mitochondria. This was further strengthened by a significant decline in mitochondrial mass, aspect ratio, and form factor, suggesting an increased fragmentation of the mitochondrial network, and consequent worsening of hyperglycemia in these patients.

A balance between the mitochondrial biogenesis and mitophagy is crucial to maintain a healthy mitochondrial pool or mitochondrial mass. Therefore, we also assessed the mitochondrial mass and observed an increase in mitochondrial mass in subjects with prediabetes. These findings indicate that augmented mitophagy in response to mild oxidative stress in subjects with prediabetes may trigger an adaptive increase in the mitochondrial mass, subsequent to enhanced mitochondrial biogenesis in order to maintain cell survival. However, subjects with NDT2DM and ADT2DM displayed attenuated mitophagy and consequent decrease in the mitochondrial mass. Collectively, these observations indicate that mitophagy precedes mitochondrial biogenesis, which is followed by an expansion of mitochondrial mass, suggesting a cross-talk between mitophagy and mitochondrial biogenesis.

A target HbA_1C_ < 7% (53 mmol/mol) is recommended by almost all the guidelines for good glycemic control (18). Our study is the first to report that besides prevention of microvascular complications, cell reparative process like mitophagy, is also sustained at HbA_1C_ < 7% (53 mmol/mol). Furthermore, HbA_1C_ > 7% (53 mmol/mol) was associated with suppressed mitophagy, irrespective of the duration of disease as observed in patients with NDT2DM and ADT2DM, thereby, strengthening the rationale of achieving target HbA_1C_ < 7% (53 mmol/mol) at any stage of the disease.

The limitations of our study include small sample size, inherent biological variability, and the lack of accomplishment of co-localization studies. Our observations of increased variation in the mitochondrial oxidative stress parameter such as SDH activity in the control group could be due to a small sample size, wider reference range for the SDH assay, and the varying stability of the enzymatic activity. Furthermore, PARKIN gene expression variability in controls and prediabetic subjects can be explained by a summation of small sample size, intra- and inter-individual variability due to a narrow range of difference in the HbA_1C_ levels across the subjects of prediabetes and healthy controls. Similarly, the protein expression variability in controls and prediabetic group can be attributed to a small sample size, biological variability as protein expression may be influenced by a variety of factors such as age, sex, and lifestyle of an individual. However, to minimize the error, both gene and protein expression studies have been normalized with their internal controls in all the experiments. Furthermore, co-localization studies could not be performed for mitophagy markers by confocal microscopy due to the limited availability of the sample as large volume of blood is required. Moreover, mitophagy cannot be measured in human PBMCs *in vivo*, and this remains a limitation for clinical studies.

In conclusion, increasing glycemic burden is associated with progressive deterioration in mitochondrial function. Sustenance of cell-reparative process like mitophagy at HbA_1C_ < 7% (53 mmol/mol) favors the rationale of achieving the HbA_1C_ below this cut-point for good glycemic control. Adaptive increase in mitophagy accompanied by increased mitochondrial mass in subjects with prediabetes may prevent or delay the progression to T2DM by limiting mitochondrial oxidative stress, mitochondrial damage, and consequent preservation of β-cell function. Thus, our data strengthen the fact that subjects with prediabetes should be efficiently screened and treated to combat the detrimental consequences of worsening hyperglycemia.

## Ethics Statement

The study was approved by the Institutional Ethics Committee (IEC) (Ref no: NK/1244/Ph.D/21478) and was performed in accordance with Helsinki Declaration. A written informed consent was obtained from the participants prior to their inclusion in the study.

## Author Contributions

SB, AB, RW, and VD contributed to the conception and design of experiments as well as the acquisition, analysis and interpretation of the data. SB drafted the manuscript, and VD, AB, and RW revised it critically for important intellectual content and approved the final version to be published. US examined and analyzed TEM findings. All the authors agreed to be accountable for the content of the work.

## Conflict of Interest Statement

The authors declare that the research was conducted in the absence of any commercial or financial relationships that could be construed as a potential conflict of interest.

## Abbreviations

ROS, reactive oxygen species; mtROS, mitochondrial reactive oxygen species; MMP, mitochondrial membrane potential; SDH, succinate dehydrogenase; TEM, transmission electron microscopy; ADA, American Diabetes Association; BMI, body mass index; WC, waist circumference; FPG, fasting plasma glucose; 2hPG, 2 h post glucose; FCP, fasting C-peptide; HOMA2-IR, homeostatic model assessment of insulin resistance; HOMA-β, homeostatic model assessment of β cell function; PINK1, PTEN-induced putative kinase 1; NIX, NIP3-like protein X; LC3, microtubule-associated protein light chain 3; LAMP-2, lysosome-associated membrane protein-2.
