# Supplementary material for: Alterations in Mitochondrial Oxidative Stress and Mitophagy in Subjects with Prediabetes and Type 2 Diabetes Mellitus
Source: Front Endocrinol (Lausanne). 2017 Dec 15;8:347. doi: 10.3389/fendo.2017.00347 (PMC5737033; doi:10.3389/fendo.2017.00347)
Supplement: Supplementary file 1 [file Table_1.DOC]

| **PRIMER** | **SEQUENCES** |
| --- | --- |
| PINK-1 | F-5’-GGGGAGTATGGAGCAGTCAC-3’  R-5’CATCAGGGTAGTCGACCAGG-3’ |
| PARKIN | F-5’-TACGTGCACAGACGTCAGGAG-3’  R-5’GACAGCCAGCCACACAAGGC-3’ |
| MFN2 | F-5’-TGATGGGCTACAATGACCAG-3  R-5’-AGCTTCTCGCTGGCATGC-3’ |
| NIX | F-5’-AAAATGAGCAGTCTCTGCCCC-3’  R-5’-TGCTGCTGTTCATGGGTAGCT-3 |
| LAMP-2 | F-5’-CGTTCTGGTCTGCCTAGTCC-3’  R-5’**-**CAGTGCCATGGTCTGAAATG-3’ |
| LC3-II | F-5’-CCACACCCAAAGTCCTCACT-3’, R-5’-CACTGCTGCTTTCCGTAACA-3’ |
| β-actin | F-5’- GGCACCCAGCACAATGAAG- 3’  R-5 -AGCTTCTCGCTGGCATGC-3’ |

**Supplementary Table I. Human-specific primer sequences for qRT-PCR**

*Abbreviations:* *PINK-1*, PTEN induced putative kinase 1; *MFN2*, Mitofusin 2; *NIX*, NIP3-like protein X; *LAMP-2,* Lysosome-associated membrane protein-2; *LC3*, Microtubule-associated protein light chain 3, *qRT-PCR*, quantitative real-time polymerase chain reaction
